# Comparative Chloroplast Genomics of Ten Collabieae Species Including Three Novel Genomes

**DOI:** 10.3390/genes16091028

**Published:** 2025-08-29

**Authors:** Shuangshuang Xie, Xingyou Jiang, Wenting Yang, Kunlin Wu, Lin Fang, Songjun Zeng, Jingjue Zeng, Lin Li

**Affiliations:** 1 Key Laboratory of South China Agricultural Plant Molecular Analysis and Gene Improvement, South China Botanical Garden, Chinese Academy of Sciences, Guangzhou 510650, China; 2 Guangdong Provincial Key Laboratory of Applied Botany, South China Botanical Garden, Chinese Academy of Sciences, Guangzhou 510650, China; 3 College of Modern Agricultural Science, Yanqihu Campus, University of Chinese Academy of Sciences (UCAS), Beijing 100049, China

**Keywords:** Collabieae, chloroplast genomes, comparative genomics, Orchidaceae, phylogenetic analysis

## Abstract

Background: Collabieae is a medium-sized group within the orchid subfamily Epidendroideae that is distributed primarily across tropical Asia. Most Collabieae species are known for their considerable ornamental and medicinal merits. However, habitat destruction and overharvesting have led to severe decline in their wild populations. Chloroplast (cp) genomes are highly valued in evolutionary studies, due to comparative conservation and accumulation of genomic variations. Elucidating the structure of chloroplast genome is instrumental in conserving genetic diversity within the Collabieae. Methods: we explored the chloroplast genome characteristics of Collabieae. We incorporated three newly sequenced genomes from species (*Acanthophippium sylhetense*, *Eriodes barbata*, and *Spathoglottis plicata*), along with seven related species. Results: all analyzed cp genomes displayed a typical quadripartite circular structure. The total lengths ranged from 157,036 bp to 158,321 bp. Each genome contained 136 genes: 88 protein-coding genes, 38 tRNA genes, eight rRNA genes, and two pseudogenes. Across the ten Collabieae species, gene number, order, orientation, GC content, and codon usage bias were highly consistent, indicative of strong sequence conservation. However, notable structural divergence was observed at the plastome junctions, alongside variations in SSR and repetitive element frequencies. Moreover, six hypervariable regions were identified. Noncoding regions exhibited higher variability compared to protein-coding regions. Phylogenetic analysis indicated that *E. barbata* forms a distinct, small branch sister to the rest of the Collabieae members. Genera *Acanthophippium* and *Spathoglottis* were sister to the remaining groups within the tribe. Conclusions: this overall phylogenetic framework aligns well with previous findings. Our study provides valuable cp genomic resources and advances evolutionary research in Collabieae.

## 1. Introduction

The tribe Collabieae is a moderately-sized orchid group, comprising 20 distinct genera. These plants are found primarily in tropical regions, with a center of diversity and endemism in Asia [1]. Most Collabieae species possess significant ornamental and medicinal values. For instance, genus *Spathoglottis* is renowned in horticulture for its unique and attractive flowers. Currently, the wild resources of these plants are declining rapidly due to habitat loss and excessive excavation. Elucidating the chloroplast genome structure is therefore essential for informing conservation efforts and preserving the genetic diversity of tribe Collabieae. Recent phylogenies [2,3] resolved a monophyletic clade of genera, characterized by single-leaved pseudobulbs. Among taxa with two- or multi-leaved pseudobulbs, *Calanthe*, *Cephalantheropsis*, and *Phaius* comprise the *Calanthe* sensu lato (s.l.) clade [4], constituting a second monophyletic group. The remaining genera (*Acanthophippium*, *Eriodes*, and *Spathoglottis*) are phylogenetically distinct from both clades. Most studies on Collabieae have primarily focused on *Calanthe* s.l. In contrast, the classification of other genera within this tribe remains controversial, and the chloroplast (cp) genomes of these species are still uncharacterized.

The three Collabieae species (*A. sylhetense*, *E. barbata*, and *S. plicata*) are all pseudobulbous geophytes, typically bearing two to five plicate leaves and occurring primarily in wet tropical regions. Despite their shared growth habit, they exhibit significant morphological divergence in floral structures, particularly in their perianth, column, and lip characteristics [5]. Species *A. sylhetense* exhibits a unique perianth structure among Collabieae: the sepals are connate at the base and connivent above, forming an urceolate tube with free apices [6]. Its distribution spans China, Laos, Malaysia, Thailand, and Vietnam. Species *E. barbata*, the sole representative of the monotypic genus *Eriodes* [1], was first described by John Lindley based on its hairy floral bracts and peduncle, with later taxonomic refinements by Robert Allen Rolfe [7]. The species is characterized by a column foot ca. 5 mm connected to a strongly recurved entire lip, via a movable joint, featuring a sagittate apex and two lateral projections. It occurs in NE India, Bhutan, Myanmar, Thailand, Vietnam, and Yunnan, China. Species *S. plicata* is widely cultivated as an ornamental orchid due to its vibrant-colored flowers and adaptability to tropical and subtropical climates [8]. The lip is T-shaped when flattened, and three-lobed, with a truncate or emarginate mid-lobe apex and two raised, hairy calli at the base. Its native range includes Malaya, Myanmar, New Guinea, Philippines, Thailand, Vietnam, China, and NE India. In China, habitat destruction and anthropogenic pressures have rendered these orchids to be classified as vulnerable, with wild populations declining rapidly [9]. Despite their ecological and horticultural significance, the chloroplast (cp) genomes of all three species remain uncharacterized. Their phylogenetic relationships require further molecular validation.

Chloroplasts are essential plant organelles, typically exhibiting maternal inheritance and possessing circular, double-stranded DNA genomes [10]. Compared to nuclear genomes, chloroplast genomes are characterized by their structural simplicity and conservation, as well as their small size and high copy numbers. These features facilitate efficient whole-genome sequencing. Recent advances in sequencing technology and cost reductions have expanded chloroplast genomics applications to phylogenetics, population genetics, and phylogeography [11]. Orchids (Orchidaceae), a highly derived family of angiosperms with significant scientific, ornamental, and medicinal value, are focal in plant phylogenetic research. Their chloroplast genomes, like those of other higher plants, adopt a quadripartite structure, spanning 148~163 kb and encoding 100~140 genes. The chloroplast genomes typically consist of one large single-copy (LSC) region; one small single-copy (SSC) region, and two inverted repeat (IRa and IRb) regions. The junctions between these regions are termed boundaries. The orchid chloroplast genome contains four boundaries, designated as JLB (LSC–IRb), JSB (SSC–IRb), JSA (SSC–IRa), and JLA (LSC–IRa). With thousands of orchid chloroplast genomes now available in NCBI databases, these data are increasingly utilized for taxonomic and phylogenetic studies within the family [12,13,14].

In our study, we sequenced and assembled three complete cp genomes of Collabieae species (*A. sylhetense*, *E. barbata*, and *S. plicata*). The newly generated cp genome sequences were then comparatively analyzed against seven previously published, complete cp genomes from the same tribe, resulting in a ten-species dataset. Using this dataset, we performed comparative genomic analyses to identify structural variations, sequence divergence, and mutation patterns. We aimed to: (1) characterize the cp genomes of these three species; and (2) elucidate genetic variation patterns across all ten genomes to verify the taxonomic status of these three species. This work delivers valuable cp genomic resources that are crucial for advancing genetics and evolutionary studies within the tribe Collabieae.

## 2. Materials and Methods

### 2.1. Material Sampling, DNA Isolation, and Sequencing

Fresh leaf samples of *A. sylhetense*, *E. barbata*, and *S. plicata* were collected from living plants in the greenhouse at the South China Botanical Garden, Chinese Academy of Sciences (SCBG, CAS 23°10.858′ N, 113°21.136′ E, 27 m). These living plants were originally collected in Yunnan, China. Voucher specimens have been deposited in the herbarium of South China Botanical Garden (IBSC), under collection numbers WTss-010 (*A. sylhetense*) xss-007 (*E. barbata*), and WTss-007 (*S. plicata*). Total genomic DNA was isolated from the leaf tissue using the Trelief Hi-Pure Plant Genomic DNA Kit (Tsingke Biological Technology Co. Ltd., Beijing, China). All processes were carried out following manufacturer’s instructions. The concentrations of the obtained DNA samples were 131.0 ng/μL, 90.8 ng/μL, and 164.5 ng/μL, respectively. Whole-genome sequencing and library construction for three species were performed on the DNBSEQ sequencing platform (Beijing Genomics Institute, BGI, Wuhan, China). The extracted DNA was fragmented via ultrasonic shearing, and fragments ranging from 300~400 bp were selected for DNBSEQ short-read library preparation. After quality assessment, the library was sequenced in paired-end (PE) 150 bp mode on the DNBseq-2000RS platform. Finally, raw sequencing reads were processed using SOAPnuke to filter low-quality sequences, generating high-quality clean data for subsequent analysis. After filtering, 1.20 × 10^7^ clean reads were obtained, respectively.

### 2.2. Complete Chloroplast Genome Assembly and Annotations

After receiving the clean data, the GetOrganelle pipeline was used to assemble the chloroplast genomes [15]. Three output graph files “gfa” were visualized and validated for correct topology using Bandage v0.8.1. Genome annotation was performed through CPGAVAS2; a based tool for plastome sequence annotation was chosen for annotating the chloroplast genome. The reference species, *Calanthe angustifolia* (NC_084139), obtained from the NCBI GenBank database, was selected. Final annotation refinements were made using CPStools [16], followed by manual correction. Complete chloroplast genome maps for *A. sylhetense*, *E. barbata*, and *S. plicata* were generated using GeSeq [17].

### 2.3. Relative Synonymous Codon Usage (RSCU), and Nucleotide Diversity Analysis

Based on the annotated chloroplast genomes, we extracted all protein-coding sequences (CDSs). To conduct codon usage bias analysis, we refined the dataset by filtering out repetitive sequences, sequences shorter than 300 bp, and sequences lacking the standard ATG start codon. Subsequently, the selected CDSs were then analyzed using an online platform (http://112.86.217.82:9929/#/tool/alltool/detail/287, accessed on 25 April 2025) to generate RSCU bar charts. RSCU values > 1 indicate codon usages were used more frequently than expected under equal usage. Following multiple sequence alignment, we conducted a sliding window analysis using the same platform. The window size was 600 bp and the step size was 200 bp.

### 2.4. Comparative Chloroplast Genomes Analysis of Ten Species from Collabieae

Our study analyzed ten species, including *Calanthe brevicornu* (OL348396), *Calanthe. nipponica* (OL348398), *Cephalantheropsis halconensis* (OK180417), *Cephalantheropsis. obcordate* (MN708351), *Phaius columnaris* (OK180421), *Phaius. mishmensis* (OK180423), and *Spathoglottis aurea* (OQ411079) with chloroplast genomes retrieved from the National Center for Biotechnology Information (NCBI), and *A. sylhetense* (PV995374), *E. barbata* (PV995375), and *S. plicata* (PV995376) that we assembled de novo. Collinearity was assessed using progressive mauve alignment in Geneious Prime 2025.0.2. We examined four chloroplast genome border junctions (LSC-IRb, IRb-SSC, SSC-IRa, IRa-LSC using CPJSdraw. Repeat sequence analysis identified four types (complement, forward, palindromic, reverse) via REPuter. The minimal repeat size was 30 bp, and the hamming distance was 3 bp. Simple sequence repeats (SSRs), including mono-, di-, tri-, tetra-, penta-, and hexanucleotide repeats were detected using MISA-web with threshold parameters of 10, 5, 4, 3, 3 for mono-, di-, tri-, tetra-, and penta-/hexanucleotide repeats, respectively.

### 2.5. Phylogenetic Analyses in Collabieae

In our study, we determined phylogenetic positions of *A. sylhetense*, *E. barbata*, and *S. plicata* within the tribe Collabieae by using the maximum likelihood (ML) method. [18]. Analysis was conducted in the IQ-Tree program [19]. A total of 16 Collabieae species were included in ML tree. Three Malaxidinae species, *Dendrobium officinale* (MN617017), *Bulbophyllum inconspicuum* (MN200377), and *Liparis auriculata* (MN200365) were designated as outgroups. First, multiple sequence alignment of all 22 taxa was performed using the MAFFT Alignment plugin in Geneious Prime. Subsequently, IQ-Tree 1.6.12 was used for phylogenetic reconstruction, and model TVM + F + R4 was selected as the best-fit substitution model, with nodal support assessed through 1000 replicates each of SH-like approximate likelihood ratio tests (SH-aLRT) and ultrafast bootstrap approximation (UFboot) [20,21]. If the values of SH-aLRT and UFboot were greater than 90%, ML tree was reliable. Finally, we employed the interactive Tree of Life (iTOL) v7 online platform [22] to visualize and annotate the phylogenetic tree of Collabieae.

## 3. Results

### 3.1. Plastome Structures of Three Collabieae Species

**Figure 1:**
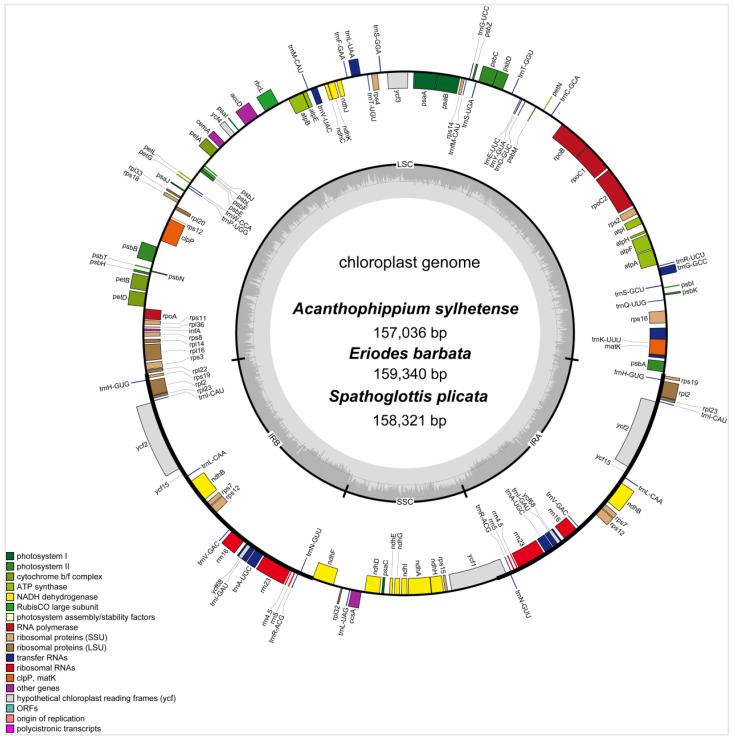
Plastome map of *A*. *sylhetense*, *E*. *barbata*, and *S*. *plicata*. All genes were annotated based on their transcription direction (clockwise inside, counterclockwise outside). Different colors represent different functions. The inner ring shows the GC (dark gray) and AT content (light gray).

The newly sequenced chloroplast genomes of *A. sylhetense*, *E. barbata*, and *S. plicata* featured a typical quadripartite structure (Figure 1), with lengths of 157,036 bp, 159,340 bp, and 158,321 bp, respectively. Each genome consisted of one large single-copy (LSC) region (86,310 bp, 87,446 bp and 86,943 bp); one small single-copy (SSC) region (17,952 bp, 18,314 bp and 18,238 bp), and two inverted repeat (IRa and IRb) regions (6387 bp, 26,790 bp and 26,579 bp each) separating the LSC and SSC regions. Their total GC content was 37.1%, 36.9%, 37.3%, respectively, with regional variations: LSC (34.9%, 34.6%, 35.0%), SSC (30.5%, 29.9%, 30.8%), and IR regions (43.1%, 43.1%, 43.3%) (Table S1). Each chloroplast genome encodes 136 genes, including 88 protein-coding genes, 38 tRNA genes, eight rRNA genes, and two pseudogenes. They share identical gene arrangements, without structural rearrangements. In their coding sequences (CDS), all *ndh* genes (A–K) are present. A total of 13 genes have cis-splicing elements, with genes *ycf3* and *clpP* each containing two introns (Figure S1). Additionally, 11 genes, including the two copies of *rpl2* and *ndhB*, each have a single intron. Each trans-splicing gene *rps12*, with three exons, was also identified (Figure S2).

### 3.2. Analysis of Repeat Structures and SSRs

**Figure 2:**
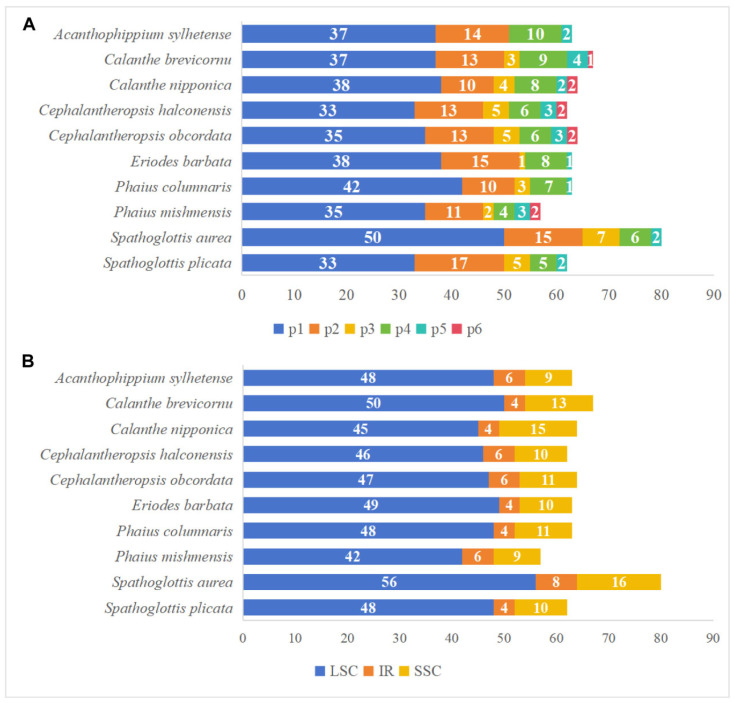
Analysis of SSRs in the plastomes of ten Collabieae species. (**A**): Number of six SSR types. (**B**): Number of SSRs located in different genomic regions.

Among the ten Collabieae species investigated, a total of 645 SSRs were found, with the number varying from 57 in *P. mishmensis* to 80 in *S. aurea*. As illustrated in Figure 2A, mononucleotide repeats were the most abundant, followed by dinucleotide, tetranucleotide, trinucleotide, and pentanucleotide repeats. Hexanucleotide repeats, however, were not detected in every Collabieae species, including *A. sylhetense*, *E. barbata*, *P. columnaris*, *S. aurea*, and *S. plicata*. Furthermore, SSR distribution was uneven across the four genomic regions. The LSC region contained the highest number of SSRs (42 to 56), followed by the SSC region (9 to 16) and the IR regions (4 to 8) (Figure 2B).

**Figure 3:**
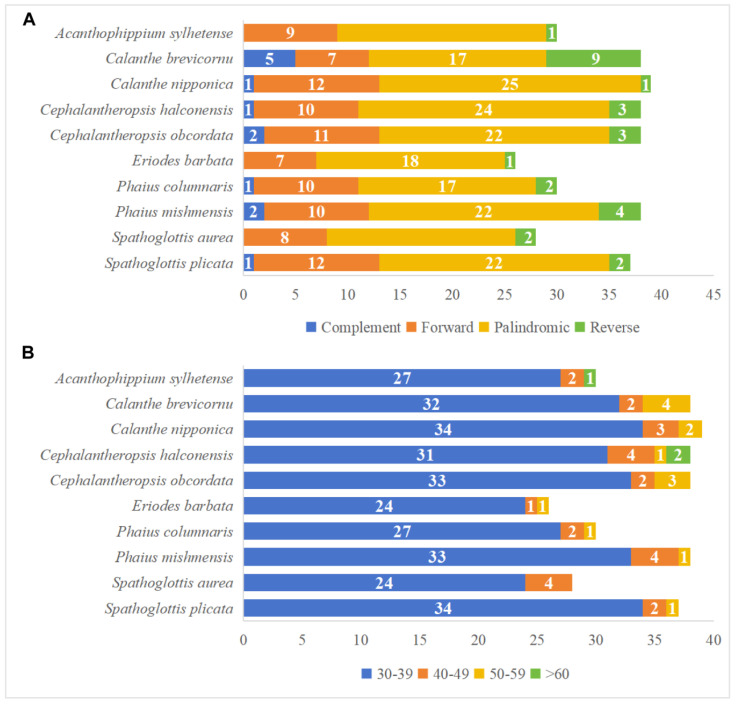
Repeat sequence analysis in ten Collabieae chloroplast genomes. (**A**): Number of four repeat types. (**B**): Number of repeated sequences identified by length.

Our study identified four categories of long tandem repeats: complementary (C), forward (F), palindromic (P), and reverse (R) repeats. The number of oligonucleotide repeats (≥30 bp) was lowest in *E. barbata* with a total of 28, while *C. nipponica* had the highest at 39. Among these species, palindromic repeats were most frequent, ranging from 17 to 22, whereas reverse repeats (R) were the least common, with zero to five instances, and were not found in *A. sylhetense*, *E. barbata*, or *S. aurea* (Figure 3A). Most detected repeats had lengths concentrated in 30–39 bp. Only *A. sylhetense* and *Ce. halconensis* had repeat sequences longer than 60 bp (Figure 3B). These findings highlight the high diversity of the ten Collabieae chloroplast genomes and offer a data foundation for further research.

### 3.3. Contraction and Expansion Analysis of IR Regions

**Figure 4:**
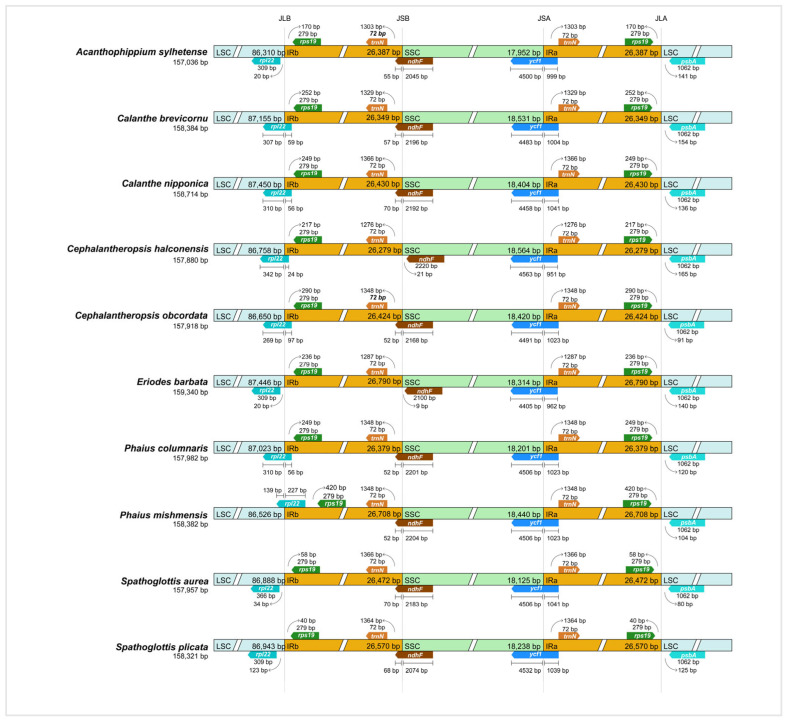
Comparison of the four boundaries (JLB, JSB, JSA, and JLA) in ten Collabieae chloroplast genomes. Arrows denote the distances (in base pairs) of genes from each junction.

In our study, a comparative analysis of the IR/SC boundaries was conducted across ten Collabieae species, revealing relatively conserved chloroplast genomes. The JLA (IRa to LSC) region is located in the intergenic spacer (IGS) between the *rps19* and *psbA* genes. The *ycf1* gene spans the JSA (SSC to IRa) boundary, extending 951 to 1041 bp into the IRa region. Simultaneously, *trnN* and *ndhF* are the two genes closest to the JSB (IRb to SSC) boundary, positioned on its left and right sides, respectively. On the contrary, the ten Collabieae chloroplast genomes exhibit several structural variations at the junctions of chloroplast genomes, as shown in Figure 4. The *rps19* gene is generally near the LSC to IRb junction (40~290 bp), except in *P. mishmensis*, where its *rps19* gene is 420 bp away. The *rpl22* genes exhibit two types: one lies entirely within the LSC region, as in *A. sylhetense*, *E. barbata*, *S. aurea*, and *S. plicata*; the other overlaps with the JLB (LSC to IRb) region, with distances in the IRb region ranging from 24 to 170 bp in *C. brevicornu*, *C. nipponica*, *Ce. halconensis*, *Ce. obcordata*, *P. columnaris*, and *P. mishmensis*. The *ndhF* gene also has two types: one is located in the regions of SSC completely, like in *Ce. halconensis* and *E. barbata*, at distances of 21 and 9 bp from IRb to the SSC region; the other extends from the JSB (IRb to SSC) region, overlapping by 52 to 70 bp.

### 3.4. The Analysis of Sequence Divergence and Mutational Hotspots

**Figure 5:**
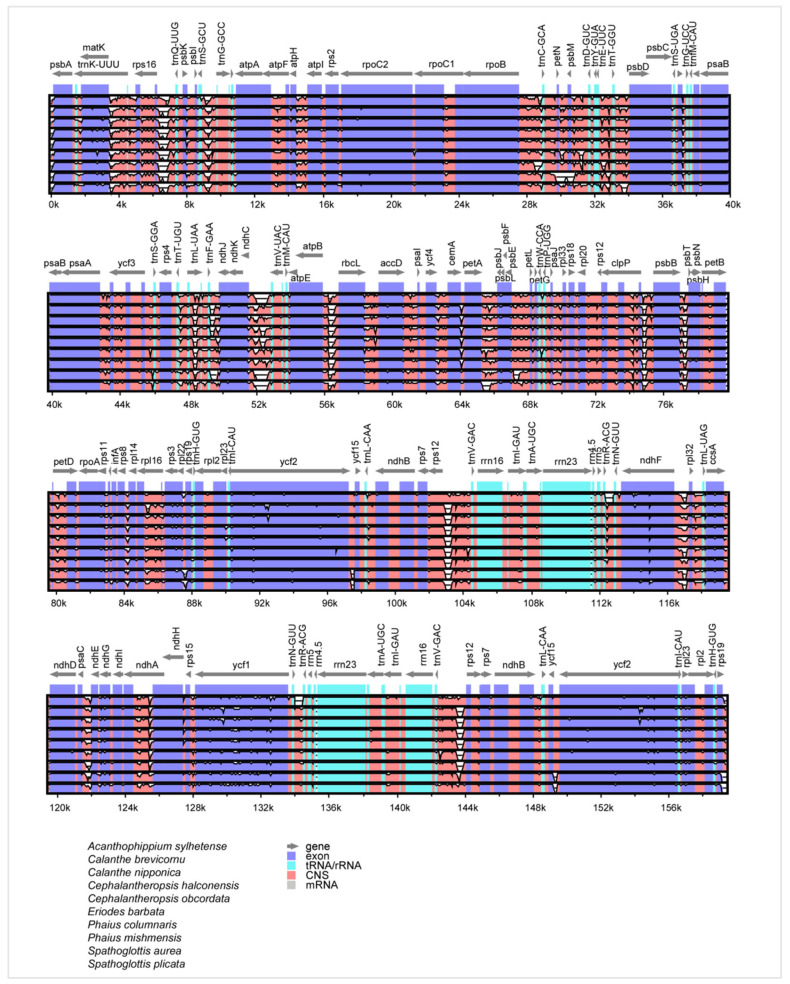
Sequence alignment of ten Collabieae chloroplast genomes. Vertical scale: identity percentage (50–100%). Horizontal axis: chloroplast genome coordinates. Genome regions are color-coded by gene, exon, tRNA/rRNA, CNS, and mRNA.

In this study, genetic variations across ten Collabieae species were analyzed and visualized using the mVISTA program (Figure 5). The results indicated that the noncoding regions were more variable than the protein-coding regions, despite conserved gene numbers, arrangements, and orientations. Notably, intergenic spacer regions exhibited particularly high genetic variability, including *trnK*-*UUU*–*rps16*, *ps16*–*trnQ*-*UUG*, *trnS*-*GCU*–*trnG*-*GCC*, *atpH*–*atpI*, *rpoB*–*trnC-GCA*, *petN*–*psbM*, *psbM*–*trnD*-*GUC*, *trnE*-*UUC*–*trnT*-*GGU*, *trnT*-*GGU*–*psbD*, *trnL*-*UAA*–*trnF*-*GAA*, *trnF*-*GAA*–*ndhJ*, *ndhC*–*trnV*-*UAC*, *atpB*–*rbcL*, *petA*–*psbJ*, *clpP*–*psbB*, *psbB*–*psbT*, *rps12*–*trnV*-*GAC*, *ndhF*–*rpl32*, *psaC*–*ndhE*, *trnV*-*GAC*–*rps12*, and *rps19*–*psbA*. Additionally, certain coding genes, such as *clpP*, *petD*, *rpl16*, *ndhA*, and *ycf1* genes displayed great variability. A clear distinction was observed between single-copy (SC) regions, which showed higher divergence than the relatively conserved inverted repeat (IR) regions. Furthermore, rRNA genes were found to be highly conserved across all examined species.

**Figure 6:**
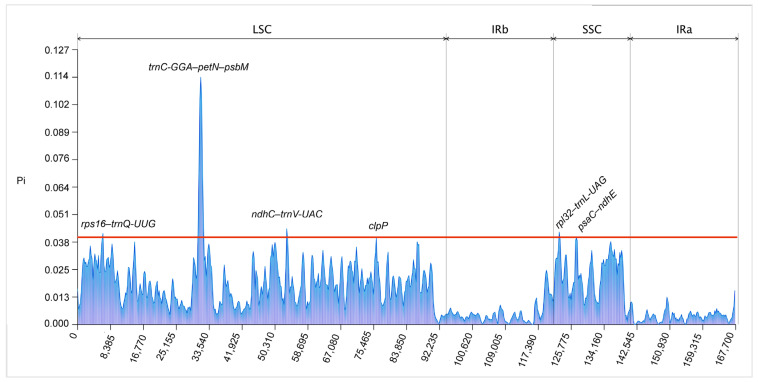
Comparison of nucleotide diversity (Pi) values across the ten Collabieae species (window length: 600 bp; step size: 200 bp).

To evaluate the genetic diversity among ten Collabieae species, we calculated nucleotide diversity values by employing a sliding window approach. The window size was set to 600 bp with a step size of 200 bp. After nucleotide diversity (Pi) analysis, six regions with high nucleotide diversity were identified, where Pi values exceeded 0.040 (Figure 6). These mutational hotspots (*rps16*–*trnQ*-*UUG*, *trnC*-*GCA*–*petN*–*psbM*, *ndhC*–*trnV*-*UAC*, *rpl32*–t*rnL*-*UAG* and *psaC*–*ndhE*) were predominantly located in noncoding regions, with the exception of *clpP*. Notably, *rps16*–*trnQ*-*UUG* also exhibited a higher degree of variability in other Collabieae species [23,24]. The Pi values in the two IR regions were found to be lower than those in the SC regions, indicating that mutation hotspots are primarily confined to the SC regions and are largely absent from the IR regions. These hotspots were unevenly distributed. Six hotspots were identified in the LSC region and two were located in the SSC region.

### 3.5. Codon Usage Analysis

**Figure 7:**
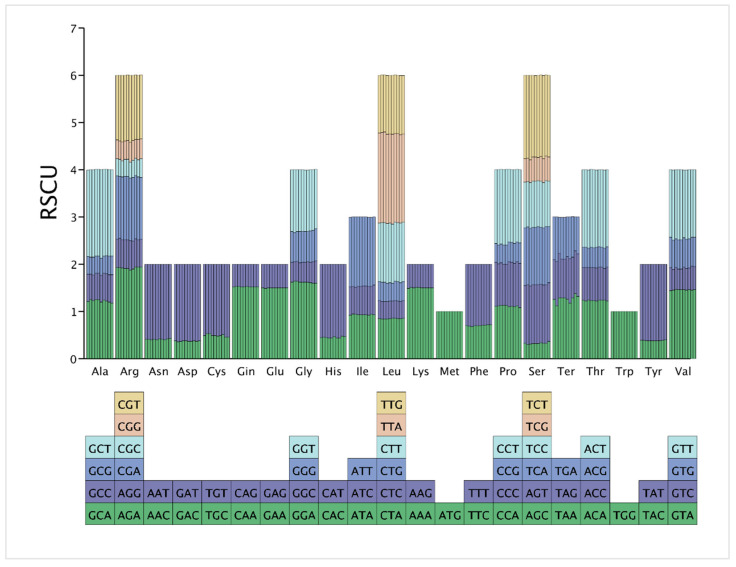
RSCU analysis for ten Collabieae species. The x-axis indicates amino acids, grouped by family. Bar height represents codon usage frequency. Colors correspond to individual codons as shown in the legend.

In this study, RSCU values for the cp genomes of ten Collabieae species were analyzed and are presented in Figure 7. After excluding the three termination codons (UAA, UAG, and UGA) and two codons (AUG and UGG), which have fixed RSCU values of 1, the RSCU analysis was performed on the remaining 59 codons. The RSCU values revealed a consistent pattern of codon preference across the genomes. Biased usage (RSCU > 1) was observed for a set of 29 codons. These codons majorities ended in A (12 codons) or T (16 codons), except TTG codons. Conversely, 30 codons were less preferred (RSCU < 1). These codons primarily ended in G (12 codons) or C (16 codons), excluding CTA and ATA codons. Notably, in *E*. *barbata*, the TTA codons (coding for Leu) had the highest RSCU value; in *C. nipponica*, the highest RSCU values were observed for TTA (Leu) and AGA (Arg) codons; in other species, the AGA codons (coding for Arg) showed the highest RSCU value.

### 3.6. Phylogenetic Analysis

**Figure 8:**
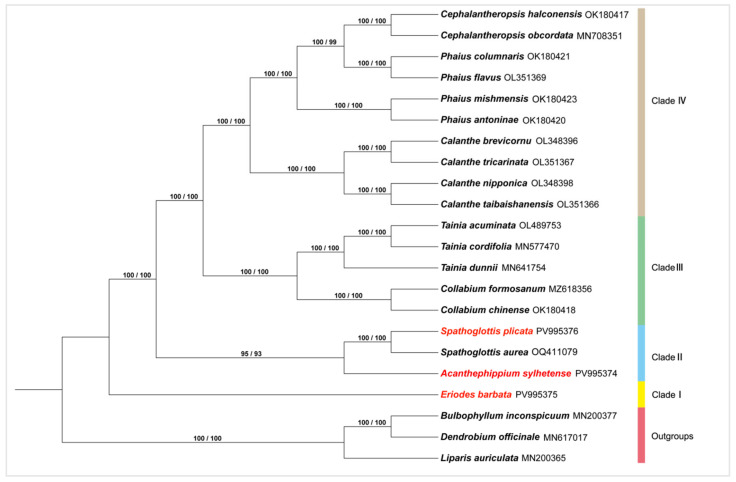
ML phylogenetic tree of Collabieae based on complete chloroplast genomes. Branch support values (SH-aLRT/UFboot, %) are indicated at the nodes. The color red denotes the newly sequenced genomes.

The phylogenetic tree of *A*. *sylhetense*, *E*. *barbata*, and *S*. *plicata* was constructed, along with 16 other species from the tribe Collabieae. Three species from the subtribe Malaxidinae (*D*. *officinale*, *B*. *inconspicuum*, and *L*. *auriculata*) were used as outgroups (Figure 8). The ML tree topology was highly robust, with nearly all nodes receiving maximum support (SH-aLRT = 100%; UFboot = 100%). The tribe Collabieae was resolved into four well-supported clades. Clade I exclusively contained *E*. *barbata*, confirming its status as a monotypic genus (SH-aLRT = 100%; UFboot = 100%). Clade II comprised genera *Spathoglottis* and *Acanthophippium* (SH-aLRT = 95%; UFboot = 93%). Clade III included genera *Tainia* and *Collabium* (SH-aLRT = 100%; UFboot = 100%). Clade IV united genera *Calanthe*, *Cephalantheropsis*, and *Phaius* (SH-aLRT = 100%; UFboot = 100%). Within clade IV, genera *Cephalantheropsis* and *Phaius* formed a sister relationship. Notably, genus *Phaius* split into two fully supported subclades (SH-aLRT = 100%; UFboot = 100%), while *Cephalantheropsis* was closely related to a subclade containing two species of *Phaius* (*P. columnaris* and *P. flavus*).

## 4. Discussion

In our study, the cp genomes of *A. sylhetense*, *E. barbata*, and *S. plicata* were characterized for the first time, and we comparatively analyzed them with seven other species from the tribe Collabieae. All ten cp genomes shared a typical quadripartite structure, with total lengths ranging from 157,036 bp to 159,340 bp, comprising a large single-copy region (LSC: 86,310 bp–87,450 bp), a small single-copy region (SSC: 17,952 bp–18,564 bp), and two inverted repeats (IR: 26,279 bp–26,708 bp). These structural features align with previous reports in orchids [25,26,27]. The overall GC content was highly conserved across species (36.6–37.3%), consistent with values reported in other Collabieae species [28,29] and within the typical range for Orchidaceae (36–37%) [30,31,32].

The cp genomes of all ten Collabieae species exhibited a high level of conservation in gene order and content. Each species was found to contain the same number of functional elements: 88 protein-coding genes (CDS), 38 tRNAs, eight rRNAs, and two pseudogenes (Table S1). Although *ycf15* and *ycf68* genes are typically categorized as non-protein-coding genes [33], our analysis revealed *ycf15* to be protein-coding, whereas *ycf68* was annotated as a pseudogene. The *ycf15* genes encode proteins, consistent with reports on *Phaius* species [24]. These observations highlight the necessity to clarify the functional roles of *ycf15* and *ycf68* in orchids.

Previous research has demonstrated that inverted repeat (IR) boundaries exhibit similarities across orchid chloroplast genomes that contain all functional *ndh* gene types [34]. Within these species, the IR regions are relatively conserved. For example, JSA junctions are consistently located approximately 1 kb downstream of the 5′ end of *ycf1* genes. Similarly, *ndhF* genes in most species occupy analogous positions near the IRb/SSC junctions. However, in *Ce. halconensis* and *E. barbata*, analyzed here, *ndhF* is translocated into the SSC region, positioned 9 bp to 21 bp from the IRb/SSC border. This relocation corresponds to the recognized phenomenon of chloroplast gene movement caused by IR boundary expansion or contraction during orchid evolution [35]. The *rpl22* genes display two distinct positional patterns at the JLB (LSC/IRb) junction, categorizing the ten species into two groups. Group I (genera *Calanthe*, *Cephalantheropsis*, and *Phaius*, which share closer evolutionary relationships) retains *rpl22* genes precisely at the LSC/IRb border. Group II (genera *Acanthophippium*, *Eriodes*, and *Spathoglottis*) shows complete relocation of *rpl22* into the LSC regions. Notably, in two *Spathoglottis* species (*S. aurea* and *S. plicata*), the *rps19* gene is closely located at the JLB junctions, at a distance of 40 to 58 bp. In contrast, it lies markedly distant (420 bp away) in *P. mishmensis*. For the remaining seven species, the distance ranges from 170 to 252 bp. Previous studies have identified three primary factors influencing chloroplast genome size variation in orchids: intergenic region variation, IR region variation, and gene loss [36]. Notably, size variation in orchid cp genomes is frequently associated with expansion and contraction dynamics in the IR regions [37,38]. Among the ten studied species, *E*. *barbata* possesses the largest chloroplast genome. This increased size is primarily attributable to the expansion of its IR regions.

Microsatellites, or SSRs, are short tandem-repeated DNA sequences with motif lengths one to six nucleotide. These markers provide abundant genetic information [39]. In this study, we detected numerous SSRs with diverse motif types and a high level of polymorphism. The number of SSRs in nine species varies from 57 to 67, while in *S. aurea*, it reaches 80. Compared to other species, *S. aurea* has a significantly higher number of SSRs (>20). This microsatellite diversity, which may be influenced by microclimatic conditions, could reflect adaptive evolution [40]. As a montane species endemic to Peninsular Malaysia [23], *S. aurea* is geographically isolated from its congeners in Borneo. Phylogenetic studies suggest that *S. aurea* may represent the closest extant ancestor of *S. microchilina* [8,41].

The deep-level evolutionary relationships of Collabieae have been unraveled by phylogenetic analyses using chloroplast genome data [42,43]. The ML phylogenetic tree confirmed Collabieae to be a monophyletic group. Within this tribe, *E. barbata* forms a distinct clade sister to all other members. Morphologically, most Collabieae species exhibit racemose inflorescences, whereas this species often has branched scapes in its upper portion. The inferred intergeneric relationships within the tribe were largely congruent with previous backbone studies [2,3], though differences arose in the placement of the genus *Acanthophippium*. One study divided Collabieae into three clades, placing the genus *Acanthophippium* within clade II, alongside the genera *Ania*, *Collabium*, *Hancockia*, *Tainia*, and *Spathoglottis*. Conversely, another study divided the tribe into nine clades, with the genus *Acanthophippium* forming a distinct branch. In our study, the genera *Acanthophippium* and *Spathoglottis* were closely related, forming a sister group. Shared morphological features include pseudobulbs bearing two to four leaves and a trilobed labellum with a clawed base in both genera. This chloroplast genome-based phylogeny yielded results that align closely with previous studies using nrITS and complete chloroplast data [3]. Phylogenetic construction using whole chloroplast genome data reduces stochastic error and typically offers greater accuracy compared to analyses based on a limited number of genes [44,45,46]. In our study, the inclusion of more species from genera *Acanthophippium* and *Spathoglottis* was constrained by sampling limitations. However, genus *Acanthophippium* is characterized by a unique campanulate (bell-shaped) perianth. This distinct feature suggests that with increased sampling efforts, the genus *Acanthophippium* is likely to be resolved as a distinct monophyletic lineage.

## 5. Conclusions

This study presents the chloroplast genomic features of three species and conducts a comparative analysis across ten species within the tribe Collabieae. The results indicate that the cp genomes of Collabieae species are generally conserved in structure, exhibiting only minor variations in gene number, order, and GC content. Nevertheless, divergence at IR junctions and SSR abundance was detected. Additionally, six mutation hotspots were identified. These hotspots serve as potential DNA barcodes for investigating phylogenetic relationships and genetic diversity. Phylogenetic analysis robustly supported the monophyly of Collabieae. Within this clade, the genus *Eriodes* formed a distinct lineage, while the genus *Acanthophippium* showed a close affinity to the genus *Spathoglottis*. Overall, these findings expand the genomic resources for Collabieae and establish a molecular foundation for evolutionary studies and species identification.

## Data Availability

The genome sequence data of *A. sylhetense*, *E. barbata*, and *S. plicata* of this study are openly available in NCBI GenBank database (https://www.ncbi.nlm.nih.gov/, accessed on 30 July 2025) under accession numbers of PV995374, PV995375, and PV995376, respectively.

## Supplementary Materials

The following supporting information can be downloaded at: https://www.mdpi.com/article/10.3390/genes16091028/s1. Figure S1: Cis-splicing gene map of chloroplast genomes in three Collabieae species; Figure S2: Trans-splicing gene *rps12* for the chloroplast genome of three Collabieae species; Table S1: Complete chloroplast genomes structural characterization of ten Collabieae species.
