# Supplementary material for: Comparative Chloroplast Genomics of Ten Collabieae Species Including Three Novel Genomes
Source: Genes (Basel). 2025 Aug 29;16(9):1028. doi: 10.3390/genes16091028 (PMC12469772; doi:10.3390/genes16091028)
Supplement: Supplementary file 1 [file genes-16-01028-s001.zip › Figure S2 Trans-splicing gene rps12 for the chloroplast genome of three Collabieae species.pdf]

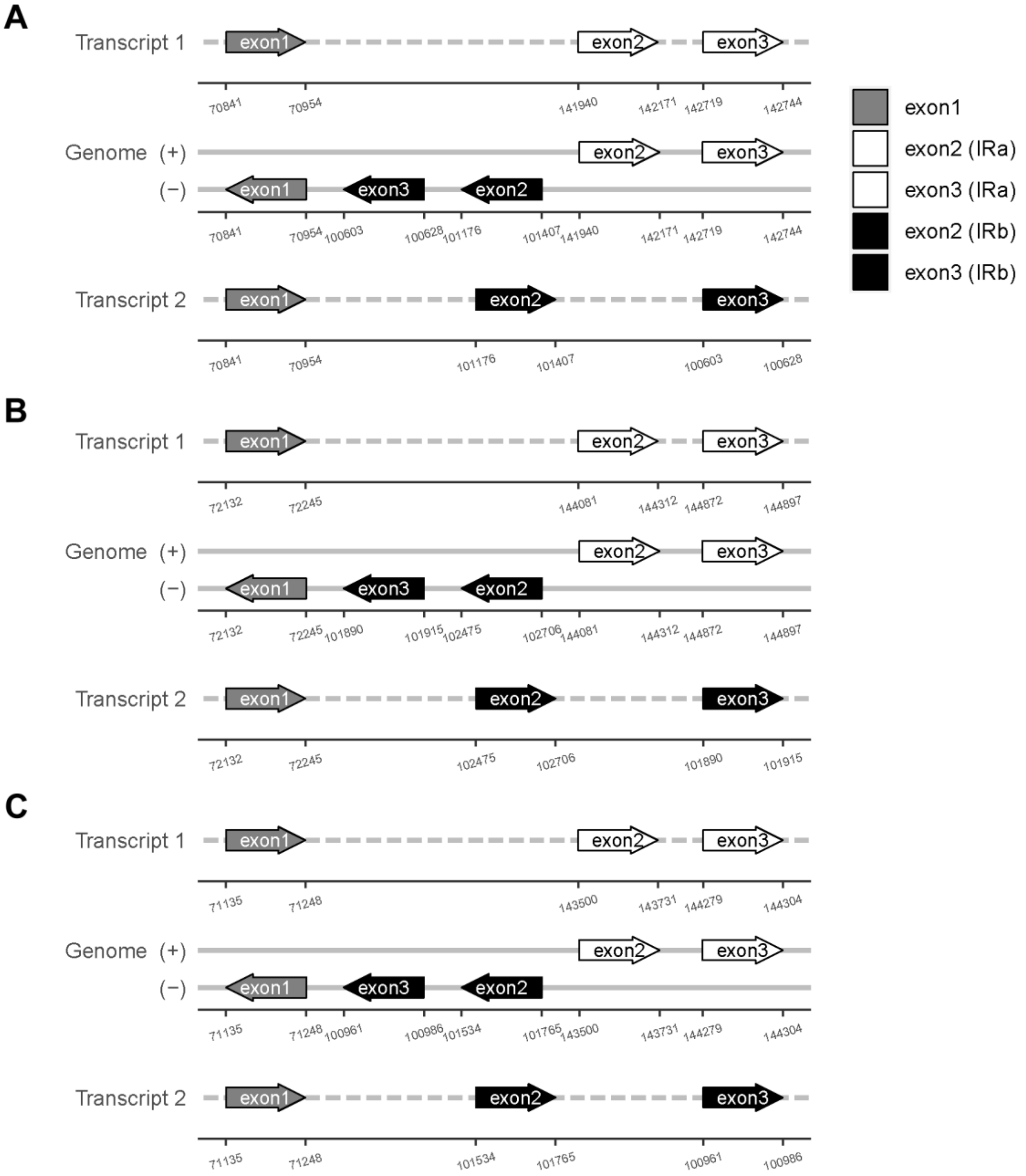

Figure S2. Trans-splicing gene *rps12* for the chloroplast genome of three Collabieae species (A: *A. sylhetense* B: *E. barbata* C: *S. plicata*). Three-panel graphs (top: transcript 1; middle: genome; bottom: transcript 2). Arrowheads indicate exonic regions of *rps12*.
