# Supplementary material for: Comparative Chloroplast Genomics of Ten Collabieae Species Including Three Novel Genomes
Source: Genes (Basel). 2025 Aug 29;16(9):1028. doi: 10.3390/genes16091028 (PMC12469772; doi:10.3390/genes16091028)
Supplement: Supplementary file 1 [file genes-16-01028-s001.zip › FIGURE~1.DOC.pdf]

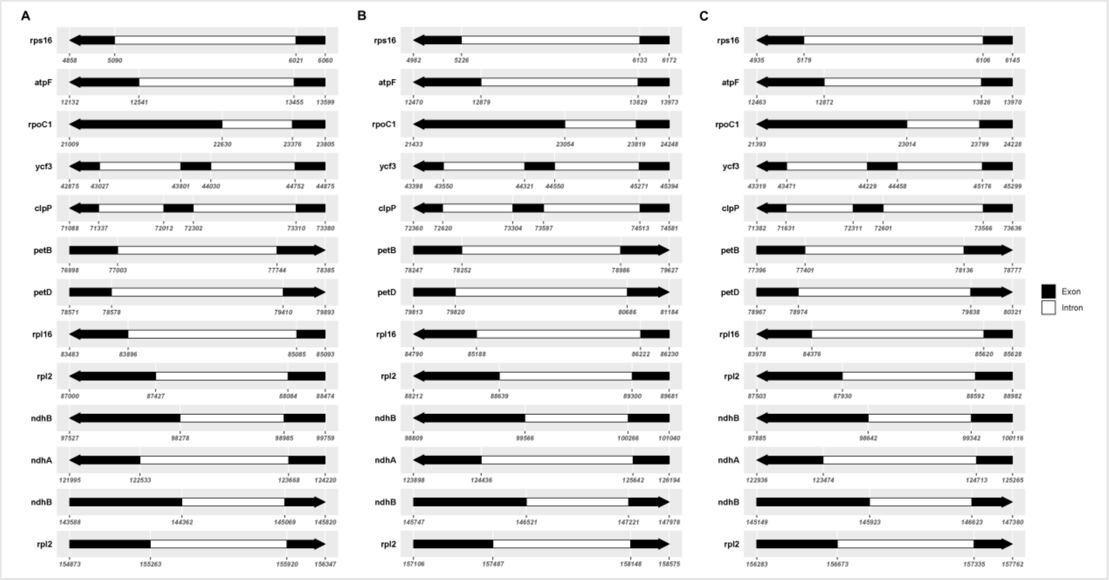

Figure S1. Cis-splicing gene map of chloroplast genomes in three Collabieae species (A: *A. sylhetense* B: *E. barbata* C: *S. plicata*). Exons denoted in black; introns in white. Arrowa indicate coding strand direction. Note: Exons/introns lengths not to scale.
