# Supplementary material for: Comparative Chloroplast Genomics of Ten Collabieae Species Including Three Novel Genomes
Source: Genes (Basel). 2025 Aug 29;16(9):1028. doi: 10.3390/genes16091028 (PMC12469772; doi:10.3390/genes16091028)
Supplement: Supplementary file 1 [file genes-16-01028-s001.zip › Table S1 Complete chloroplast genomes structural characterization of ten Collabieae species.pdf]

Table S1. Complete chloroplast genomes structural characterization of ten Collabieae species

Species	Accession	length（bp）				GC（%）				Gene Number				
		total	LSC	SSC	IR	total	LSC	SSC	IR	Total	PCG	tRNA	rRNA	Pseudogenes
A. sylhetense	PV995374	157,036	86,310	17,952	26,387	37.1	34.9	30.5	43.1	136	88	38	8	2
C. brevicornu	OL348396	158,384	87,155	18,531	26,349	36.7	34.3	29.6	43.1	136	88	38	8	2
C. nipponica	OL348398	158,714	87,450	18,404	26,430	36.6	34.2	29.7	43.0	136	88	38	8	2
Ce. halconensis	OK180417	157,880	86,758	18,564	26,279	36.8	34.5	29.8	43.2	136	88	38	8	2
Ce. obcordata	MN708351	157,918	86,650	18,420	26,424	36.8	34.5	29.7	43.1	136	88	38	8	2
E. barbata	PV995375	159,340	87,446	18,314	26,790	36.9	34.6	29.9	43.1	136	88	38	8	2
P. columnaris	OK180421	157,982	87,023	18,201	26,379	36.9	34.5	30.2	43.0	136	88	38	8	2
P. mishmensis	OK180423	158,382	86,526	18,440	26,708	36.9	34.7	30.0	43.0	136	88	38	8	2
S. aurea	OQ411079	157,957	86,888	18,125	26,472	37.3	35.0	30.7	43.2	136	88	38	8	2
S. plicata	PV995376	158,321	86,943	18,238	26,570	37.3	35.0	30.8	43.3	136	88	38	8	2
